# Survey of CAMHS clinicians about their experience of remote consultation: brief report

**DOI:** 10.1192/bjo.2020.160

**Published:** 2021-01-13

**Authors:** Anupam Bhardwaj, Anna Moore, Rudolf N. Cardinal, Carol Bradley, Lauren Cross, Tamsin J. Ford

**Affiliations:** Cambridgeshire & Peterborough NHS Foundation Trust, UK; Cambridgeshire & Peterborough NHS Foundation Trust, UK; and Department of Psychiatry, University of Cambridge, UK; Cambridgeshire & Peterborough NHS Foundation Trust, UK; and Department of Psychiatry, University of Cambridge, UK; Cambridgeshire & Peterborough NHS Foundation Trust, UK; Department of Psychiatry, University of Cambridge, UK; Cambridgeshire & Peterborough NHS Foundation Trust, UK; and Department of Psychiatry, University of Cambridge, UK.

**Keywords:** Covid-19, remote consultation, CAMHS, service delivery, digital health

## Abstract

The Covid-19 crisis necessitated rapid adoption of remote consultations across National Health Service (NHS) child and adolescent mental health services (CAMHS). This study aimed to understand practitioners’ experiences of rapid implementation of remote consultations across CAMHS in one NHS trust in the east of England. Data were collected through a brief questionnaire documenting clinicians’ experiences following remote delivery of services. The questionnaire began before ‘lockdown’ and focused on assessment consultations (*n* = 102) as part of a planned move to virtual assessment. As the roll-out of remote consultations was extended at lockdown, we extended the questionnaire to include all remote clinical contacts (*n* = 202). Despite high levels of initial concern, clinicians’ reports were positive overall; importantly, however, their experiences varied by team. When restrictions on face-to-face working are lifted, a blended approach of remote and face-to-face service delivery is recommended to optimise access and capacity while retaining effective and safe care.

The Covid-19 crisis necessitated the rapid adoption of remote consultations across the National Health Service (NHS), including in child and adolescent mental health services (CAMHS).^1^ Although there is a growing evidence base describing the effectiveness of video consultation for CAMHS, questions remain regarding effectiveness and acceptability, as well as how to address engagement, risk management and attrition.^2,3^ We lack consensus on implementation, so adoption in many countries (including the UK) has been slow.^1^ Cultural, organisational, and technological challenges seem prominent.^4^ We need more information about the clinical settings and patient groups for whom remote consultation is most appropriate. In CAMHS, the therapeutic alliance greatly influences treatment outcomes,^5^ and implementation of remote consultations is hampered by clinicians’ concerns about safety, effectiveness, technological challenges and rapport with patients.^3,6^

We lack information about clinicians’ experience of remote healthcare delivery with which to inform service improvements,^7^ so it is imperative that we learn from the current rapid implementation of remote consultations. We aimed to understand practitioners’ experience across CAMHS in one NHS trust in the east of England, to inform subsequent platform development and service delivery.

## Method

As this was a formal service evaluation, ethical approval was not required, but we provided clinicians with information sheets that explained our aims and informed them that their responses would be analysed for publication inside and outside the service.

We defined remote delivery as the use of telephone calls or videoconferencing for patient- or carer-facing clinical activity. We used a survey of clinicians as an initial approach to gather feedback from clinicians on their experience of remote consultations. Practitioners from all locality-based community teams and two trust-wide specialist teams (eating disorders and substance misuse) were invited to complete the survey (available from the authors on request) using Microsoft Forms after each consultation. Initially, the questionnaire focused mainly on ‘assessment’ as part of a planned move to virtual assessment that was expedited owing to Covid-19-related restrictions on face-to-face appointments (*n* = 102; 19 March 2020 to 1 April 2020). After feedback from clinicians, very shortly after lockdown we modified the questionnaire to cover all types of clinical consultations (*n* = 202; 2 April 2020 to 23 May 2020). We report findings from both versions of the survey, with collated responses where possible.

## Results

We were able to calculate an approximate response rate of 25% of consultations conducted during the survey period, based on the data available. The number of remote contacts was estimated from the total contacts (*n* = 1226) recorded in electronic case notes. We assumed that all contacts were remote contacts, as electronic case records did not differentiate, and received a mean of 3.5 responses per member of clinical staff (*n* = 87).

As shown in Table 1, 21% of responses related to assessments, 34% to reviews and 44% to treatment, while 68% of remote consultations were conducted over the telephone and 31% via videoconference. Of the two modalities, patients preferred the telephone. There was no significant difference in clinicians’ views with respect to rapport or safeguarding assessment. The mode used most commonly reflected patient choice (48%), compared with clinician choice (18%) or therapeutic need (34%). Only 15% of remote assessments were estimated to take longer than if conducted face-to face, whereas 24% were perceived to have been quicker and 61% to have been the same duration. Twenty-eight per cent of respondents reported technical difficulties. There was no effect on rapport for 63% of consultations, whereas a negative or positive effect was reported for 26% and 10%, respectively. Practitioners were mostly confident that they were able to perform safeguarding procedures and risk assessments. Clinicians reported that they were able to see the young person alone in three-quarters of cases when this was considered necessary, although this varied by team.

**Table 1:** Illustration of responses to survey questions and results of statistical tests

	Teams						Modality			
Variable description	Substance misuse	General CAMHS	Neurodevelopmental	Eating disorders	All teams	Statistics	Telephone	VC	All *n* = 303^b^	Statistics
Frequency reported^a^	*n* = 19 (6%)	*n* = 180 (59%)	*n* = 80 (26%)	*n* = 25 (8%)			*n* = 208 (69%)	*n* = 95 (31%)		
Contact type (*n* = 304)^c^										
Assessment	11 (58%)	39 (22%)	11 (14%)	<10 (16%)	65 (21%)	χ^2^ = 28.07 (d.f. = 6), *P* < 0.0001, Cramer's V = 0.21	41 (20%)	23 (24%)	64 (21%)	χ^2^ = 6.28 (d.f. = 2), *P* < 0.043, Cramer's V = 0.14
review	<10 (11%)	55 (31%)	34 (43%)	13 (52%)	104 (34%)		81 (39%)	23 (24%)	104 (34%)	
Treatment	<10 (32%)	86 (48%)	35 (44%)	<10 (32%)	135 (44%)		86 (41%)	49 (52%)	135 (44%)	
Modality (*n* = 304)^c^										
Telephone	14 (74%)	125 (69%)	63 (79%)	<10 (24%)	208 (68%)	χ^2^ = 28.07 (d.f. = 6), *P* < 0.0001, Cramer's V = 0.21	na	na	na	na
VC	<10 (26%)	54 (30%)	17 (21%)	19 (76%)	95 (31%)		na	na	na	
F2F	0 (0%)	<10 (<10%)	0 (0%)	0 (0%)	<10 (<10%)		na	na	na	
Modality reason (*n* = 202)^c^										
Clinician choice	<10 (35%)	22 (20%)	<10 (<10%)	<10 (17%)	36 (18%)	χ^2^ = 16.65 (d.f. = 6), *P* = 0.01, Cramer's V = 0.20	21 (16%)	15 (21%)	36 (18%)	χ^2^ = 20.89 (d.f. = 2), *P* < 0.0001, Cramer's V = 0.32
Patient choice	11 (64%)	54 (49%)	26 (46%)	<10 (33%)	97 (48%)		76 (59%)	20 (27%)	96 (48%)	
Therapeutic need	0 (0%)	35 (32%)	25 (45%)	<10 (50%)	69 (34%)		31 (24%)	38 (52%)	69 (34%)	
Technical problems (*n* = 304)^c^										
No	17 (89%)	125 (69%)	63 (79%)	13 (52%)	218 (72%)	χ^2^ = 10.15 (d.f. = 3), *P* = 0.017, Cramer's V = 0.18	162 (78%)	55 (58%)	217 (72%)	χ^2^ = 12.82 (d.f. = 1), *P* < 0.0001, Cramer's V = 0.21
Yes	<10 (11%)	55 (31%)	17 (21%)	12 (48%)	86 (28%)		46 (22%)	40 (42%)	86 (28%)	
Time taken for contact (*n* = 102)^c^										
<45 min	<10 (50%)	42 (60%)	18 (75%)	0 (0%)	61 (60%)	χ^2^ = 16.27 (d.f. = 6), *P* = 0.012, Cramer's V = 0.28	57 (71%)	<10 (18%)	61 (60%)	χ^2^ = 20.57 (d.f. = 2), *P* < 0.0001, Cramer's V = 0.45
45–60 min	0 (0%)	13 (19%)	<10 (21%)	<10 (43%)	21 (21%)		11 (14%)	10 (45%)	21 (21%)	
>60 min	<10 (50%)	14 (20%)	<10 (<10%)	<10 (57%)	20 (20%)		12 (15%)	<10 (36%)	20 (20%)	
Relative time taken (*n* = 202)^c^										
Less time compared with F2F	<10 (24%)	29 (26%)	14 (25%)	<10 (11%)	49 (24%)	χ^2^ = 4.44 (d.f. = 6), *P* = 0.62, Cramer's V = 0.10	43 (34%)	<10 (8%)	49 (24%)	χ^2^ = 16.25 (d.f. = 2), *P* < 0.0001, Cramer's V = 0.28
More time compared with F2F	<10 (17%)	18 (16%)	<10 (14%)	<10 (6%)	30 (15%)		17 (13%)	13 (18%)	30 (15%)	
Similar time compared with F2F	10 (59%)	64 (58%)	34 (61%)	15 (83%)	123 (61%)		68 (52%)	54 (74%)	122 (61%)	
Clinicians’ views of the modalities’ effect on rapport (*n* = 304)^c^										
Negative	<10 (18%)	31 (28%)	12 (21%)	<10 (39%)	53 (26%)	χ^2^ = 33.85 (d.f. = 6), *P* < 0.0001, Cramer's V = 0.29	33 (26%)	20 (27%)	53 (26%)	χ^2^ = 5.91 (d.f.=2), *P* = 0.05, Cramer's *V* = 0.17
No influence	<10 (35%)	68 (61%)	43 (77%)	11 (61%)	128 (63%)		87 (68%)	41 (56%)	128 (64%)	
Positive	<10 (47%)	12 (11%)	<10 (<10%)	0 (0%)	21 (10%)		<10 (<10%)	12 (16%)	20 (10%)	
Was it possible to assess safeguarding adequately? (*n* = 102)^c^										
No	0 (0%)	<10 (<10%)	<10 (13%)	<10 (14%)	10 (10%)	χ^2^ = 0.67 (d.f. = 3), *P* = 0.88, Cramer's V = 0.08	<10 (<10%)	<10 (14%)	10 (10%)	χ^2^ = 0.47 (d.f. = 1), *P* = 0.05, Cramer's V = −0.07
Yes	<10 (100%)	63 (91%)	21 (88%)	<10 (86%)	92 (90%)		73 (91%)	19 (86%)	92 (90%)	
Was it possible to speak to the Children and Young People alone if required? (*n* = 304)^c^										
No	0 (0%)	32 (18%)	23 (29%)	<10 (28%)	62 (20%)	χ^2^ = 40.65 (d.f. = 6), *P* < 0.0001, Cramer's V = 0.26	46 (22%)	16 (17%)	62 (20%)	χ^2^ = 11.64 (d.f. = 2), *P* = 0.003, Cramer's V = 0.20
Not applicable	0 (0%)	24 (13%)	24 (30%)	10 (40%)	58 (19%)		29 (14%)	29 (31%)	58 (19%)	
Yes	19 (100%)	124 (69%)	33 (41%)	<10 (32%)	184 (61%)		133 (64%)	50 (53%)	183 (60%)	
Clinicians’ views on Children and Young People and families’ perceptions of remote consultation (*n* = 202)^c^										
Negative	<10 (<10%)	41 (23%)	11 (14%)	(0%)	34 (17%)	χ^2^ = 18.87 (d.f. = 6), *P* < 0.004 Cramer's V = 0.22	26 (20%)	<10 (11%)	34 (17%)	χ^2^ = 15.36 (d.f. = 2), *P* < 0.0001, Cramer's V = 0.28
Neutral	<10 (29%)	82 (46%)	51 (64%)	15 (61%)	103 (51%)		73 (57%)	29 (40%)	102 (51%)	
Positive	12 (65%)	57 (32%)	17 (21%)	10 (39%)	65 (32%)		29 (23%)	36 (49%)	65 (32%)	

F2F, face-to-face; na, not applicable; VC, videoconference.

a. Frequencies and percentages have been rounded to one significant figure (the nearest whole number).

b. *N* = 303 for modality as one face-to-face contact was excluded as it was not possible to perform analysis.

c. *N* = 304 results were reported by collating responses from versions 1 and 2 of the questionnaire. *N* = 102 results were reported from version 1 only. *N* = 202 results were reported from version 2 only.

Other experiences varied by team (Table 1), including the type of consultation, the modality chosen and the reason provided for this choice. The substance misuse team and eating disorder team reported opposite effects on rapport: negative for 18% and 39% of their consultations and positive for 47% and 0%, respectively. Two-thirds (65%) of reports from the substance misuse team suggested that young people and families perceived the remote consultations positively, whereas those from the eating disorders and neurodevelopmental pathways were mostly neutral (61% and 64%, respectively) and those from locality teams were most likely to be negative (23%).

## Discussion

Our findings provide valuable insights into CAMHS clinicians’ experiences of remote consultations during the Covid-19 lockdown. Clinicians’ reports were positive overall in our survey; importantly, however, their experience varied by team. CAMHS’ clientele includes many children, young people and families for whom remote consultation is a viable and perhaps even preferred option. For most consultations, meeting remotely did not take more time or adversely affect rapport, safeguarding or risk assessment. There were, however, clear differences between teams and, notably, none of our participating teams reported a wholly positive experience. Differences between teams are likely to reflect variation in clinical needs but also team culture and experience, and a challenge remains to determine for whom, and in which circumstances, remote consultation is more or less effective than face-to-face consultation.^8^ Reduced travel time for clinicians as well as for families may increase capacity if clinical effectiveness can be preserved.

Our survey was small and used a bespoke questionnaire, and we lacked comparison data on rapport, safeguarding and risk management arising from face-to-face consultations. Data were collected from one NHS trust in a particular region of England, during a pandemic, and for only one-quarter of consultations, although the response rate may have been underestimated given that there were probably a few face-to-face consultations that still had to go ahead for clinical reasons. Our findings may not therefore be generalisable to other areas or circumstances. In particular, clinicians were not only offering virtual consultations but were doing so from home, which is a different experience to working from a team base, where colleagues from the multidisciplinary team would be more readily available to discuss concerns and provide support. Given the lack of evidence and high levels of anxiety about the effects of remote consultations, these preliminary findings are important and reassuring; however, others may wish to replicate and expand them.

The current restrictions on face-to-face working have greatly increased exposure to remote consultations, which given, our more positive than expected reports from clinicians, could have been because clinicians were able to adapt their behaviour and communication style.^9^ This enforced move to remote practice is likely to improve confidence as well as willingness to experiment with remote modes of service delivery.

## Future directions

Some interventions and formal assessments may need adaptation for remote consultations, and for others it may not be possible, e.g. the autism diagnostic observation schedule (ADOS). The limited available evidence should encourage clinicians and researchers to explore this further.^10^ New research for treatment evaluation should incorporate remote consultations or a blended approach in study designs to gain information on the effectiveness of remote delivery. We should identify clinicians’ training needs and determine how to support those among the populations that we serve who experience problems with access or engagement, or raise safeguarding concerns. Mixed-methods studies, including surveys, ethnographic observations and focus groups, with input from young people, parents and referrers, could be used to co-produce guidelines on how to optimise virtual consultation and treatment, as well as how to manage engagement, risk and safeguarding. It is critical that we learn the lessons of what works for whom, so that we can retain the benefits and mitigate the risks of virtual treatment, particularly given the challenge that CAMHS face in meeting demand. Economic evaluations of whether remote working adds to efficiency are also needed, and the influence of remote working on team dynamics and culture needs further evaluation. A blended approach of remote and face-to-face service delivery in different combinations, tailored to clinical need, may optimise access and capacity while retaining effective and safe care.

## Data Availability

The data that support the findings of this study are available from the corresponding author (L.C.) upon reasonable request.
